# C.487C>T mutation in PAX4 gene causes MODY9: A case report and literature review

**DOI:** 10.1097/MD.0000000000032461

**Published:** 2022-12-23

**Authors:** Di Zhang, Congli Chen, Wenli Yang, Yurong Piao, Li Ren, Yanmei Sang

**Affiliations:** a Department of Pediatric Endocrinology, Capital Institute of Pediatrics, Beijing, China; b Department of Pediatric Endocrinology, Genetic, and Metabolism, Beijing Children’s Hospital, Capital Medical University, National Center for Children’s Health, Beijing, China.

**Keywords:** MODY, monogenic diabetes, PAX4 gene

## Abstract

Maturity-onset diabetes of the young (MODY) is a group of autosomal dominant monogenic diabetes mellitus with a wide range of clinical manifestations that require distinct treatment strategies. MODY9 (OMIM # 612225) is a rare type of MODY, caused by a mutation in the Paired box gene 4 (PAX4).

**Patient concern:**

A 19-months boy was admitted to the department of endocrinology at Beijing Children’s Hospital due to excessive water drinking, polyuria for over half a month, and wheezing for 3 days.

**Diagnose:**

The whole-exon sequencing analysis demonstrated that the child carried the heterozygous missense mutation of c.487>T in the 7th exon region of PAX4 gene and diagnosed MODY9.

**Intervention:**

The patient was treated with fluid therapy, ketosis correction, insulin, and anti-infection treatment.

**Outcomes:**

After 17 days in the hospital, the blood glucose levels remained stable and the patient was discharged.

**Lessons:**

In Chinese children, the heterozygous mutation of c.487C>T in the PAX4 gene can lead to the occurrence of MODY9.Gene sequencing analysis is of great significance in the diagnosis and classification of MODY.

**What is new?**

• This is the first case diagnosed with MODY9 reported in China.
• A de novo heterozygous c.487C>T mutation in the PAX4 gene was described.

## 1. Introduction

Maturity-onset diabetes of the young (MODY) is a group of autosomal dominant monogenic diabetes. It accounts for about 1% to 5% of all types of diabetes.^[1,2]^ MODY is classified into 14 types (MODY1-14) according to the type of mutated genes. Among them, the most common types in the Chinese population are MODY1, MODY2, and MODY3.^[3,4]^ Typical cases of MODY are characterized by early onset before the age of 25, negative pancreatic autoantibodies, islet β-cell dysfunction, and normal serum C-peptide levels. Since clinical manifestations are not indicative of diagnosis, genetic testing is crucial to accurate diagnosis and timely treatment.

MODY9 is a rare type of MODY caused by a mutation in Paired box gene 4 (PAX4), first reported in 2007.^[5]^ PAX4, a member of the PAX transcription factor family, plays an important role in the differentiation of pancreatic β-cells as a key regulator of early pancreatic development at the embryonic stage.^[6]^ To the best of our acknowledge, there is no confirmed case of MODY9 in China.

Herein, we present the clinical and genetic features of a 1-year-old Chinese child diagnosed MODY9 with a novel heterozygous c.487C>T mutation in the PAX4 gene and further discuss the relevant literature to raise clinicians’ awareness of this disease and suggest approaches to precisely diagnose and manage MODY9.

## 2. Case presentation

The proband was male, aged 19 months. He was delivered at the 39th gestational week by cesarean section. Birth weight and length were 3.35 kg and 51 cm, respectively. The neuromotor development was normal from birth to date. The patient had no family history of diabetes.

More than half a month before admission, the patient developed unprovoked polyuria and polydipsia. 2 days before admission, the child suffered from severe wheezing with Kussmaul breathing and poor general condition. His random blood glucose levels were 40 mmol/L, and his urine ketone body was positive. On arrival at the emergency unit, the intravenous blood glucose level was 20.74 mmol/L, and blood gas analysis resulted in pH 7.07, PCO2 17.5 mm Hg, BE – 23.5 mmol/L, then the patient was admitted to the endocrinology department for further treatment.

### 2.1. Physical examination on admission

Patient’s temperature was 37℃, respiration rate was 37 times/min, pulse was 150 times/min, and blood pressure was 116/62 mm Hg. The patient’s weight was 12 kg with normal physical development. He was in poor general condition, with a decreased level of consciousness, no tears when crying, an anemic appearance, Kussmaul respiration, and a fruity breath odor. His lips and skin were dry with slightly low skin elasticity. The right upper lung presented with moist carackles. The upper extremities were cool to the elbows, while the lower extremities were cool to the knees, with a dilated blood vessel pattern on the limbs and a CRT of 3 seconds.

### 2.2. Laboratory and imaging results on admission.

The blood sugar was 21.69 mmol/L (3.9–6.1 mmol/L), and glycosylated hemoglobin was 11.1% (4–6%). At the 0th minute, the insulin level was 0.56 μIU/mL (6.0–27.0 μIU/mL), C-peptide level was 0.09 ng/mL (1.1–5.0 ng/mL). Anti-islet cell and anti-glutamate decarboxylase antibodies were negative. Blood gas analysis repeated: pH 7.06, PCO2 15mm Hg, PO2 74 mm Hg. Urine routine analyses showed that the glucose was 4+, ketone was 3+, and the protein was 1+. Cortisol and thyroid function tests are normal. Chest X-ray showed slightly more texture and fuzziness in the lungs with a patchy shadow on the right upper lung. Abdominal ultrasound showed no abnormalities.

The patient was diagnosed with type 1 diabetes, diabetic ketoacidosis, and pneumonia. After fluid therapy, ketosis correction, insulin, and anti-infection treatment, the patient gradually recovered. After 17 days in the hospital, blood glucose levels remained stable and the patient was discharged with a total insulin dosage at discharge was 7.5 IU, equivalent to 0.625 IU/kg/d.

### 2.3. Results of genetic analysis

2mL of peripheral venous blood was collected (EDTA anticoagulant), and genomic DNA was extracted using the QIAamp whole blood DNA extraction kit (Qiagen, Germany). Whole-exon sequencing analysis after informed consent from the patient’s parents was conducted.

**Figure 1.**
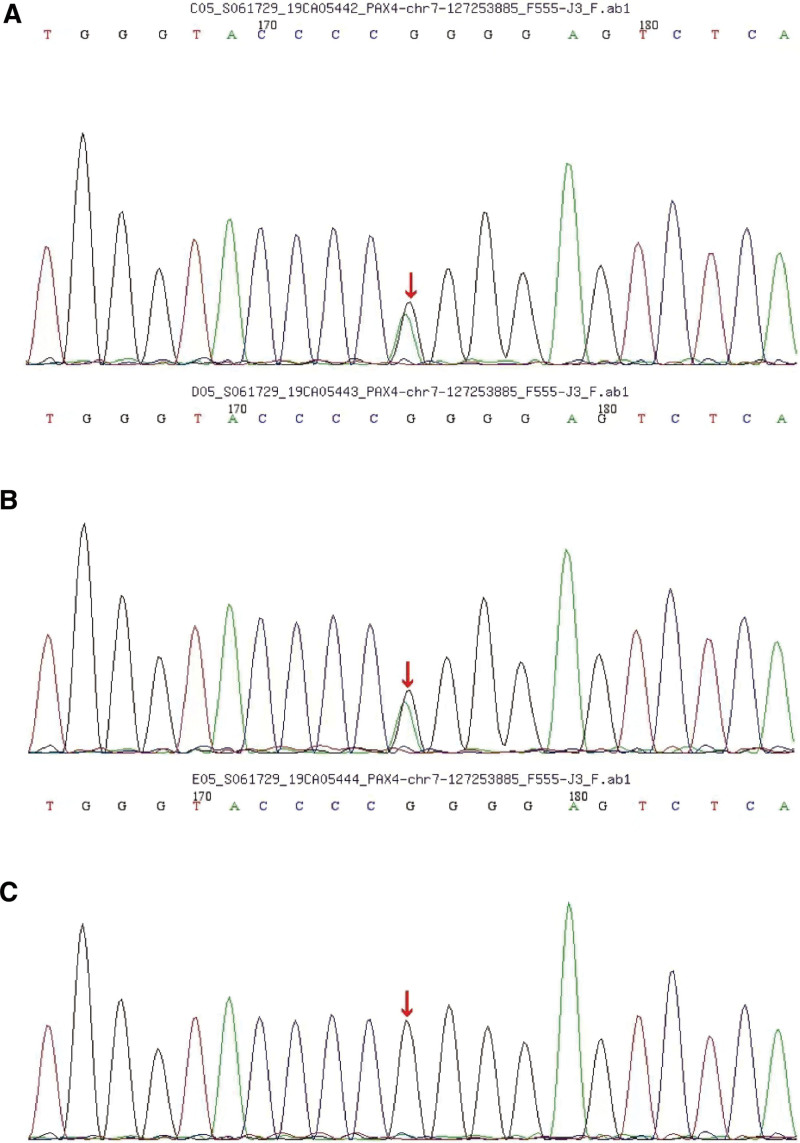
PAX4 gene sequencing of the patient and his parents. (A) Genetic sequencing results revealed that the patient carried a rare c.487C>T heterozygous mutation in the 7th exon region of the PAX4 gene. (B) The patient’s father carried the same heterozygous mutation as the patient. (C) The patient’s mother presented with a normal genotype and did not carry the c.487C>t heterozygous mutation. PAX4 = paired box gene 4.

The genetic sequencing results revealed that the patient carried a rare c.487C>T heterozygous mutation in the 7th exon region of the PAX4 gene (Fig. 1). This mutation led to an R163W variation in the amino acid sequence. The patient’s father carried the same heterozygous mutation while the mother presented with a normal genotype, suggesting that the mutation could be autosomal dominant. The estimated frequency of the mutation in the normal population database was 0.0001, which is a low-frequency mutation. There were no related reports of this mutation in the HGMD database. SIFT, PolyPhen_2, Mutation Taster, GERP++, and REVEL respectively predicted this mutation as harmful, harmful, benign, harmful, and benign. According to the ACMG guideline, the mutation was preliminarily determined to be of unknown clinical significance.

## 3. Discussion and conclusion

PAX4 is located at 7q32.1, contains 10 exons and 9 introns with a molecular weight of 7851 bp, and encodes the paired cassette protein PAX4, containing potential DNA binding structural domains.^[6]^ PAX4 protein, expressed mainly in islet pancreatic cells, regulates the generation of islet progenitor cells and the differentiation of islet *β*-cells and islet *δ*-cells. Mutation in PAX4 can disrupt PAX4 protein targeting ability, an increase in alpha cells, and an increase in glucagon secretion, resulting in elevated blood glucose.^[6]^

Extremely few cases of MODY9 have been reported to date. In 2007, Nattachet et al performed DNA direct sequencing techniques on 46 suspected MODY patients (all of Thai origin) to detect the pathogenic gene and identified a missense mutation(R164W) and a shear mutation(IVS7-1) in the PAX4 gene in two diabetic families, defined as MODY9.^[5]^ In 2011, Jo et al^[7]^ reported a Japanese MODY9 family carrying a deletion mutation in the third exonic region of PAX4, C374-412del39. In 2018, Pezzilli et al^[8]^ reported a MODY9 Italian family with a PAX4 c.593C>T (A198V) missense mutation in four diabetic patients. Three more missense mutations of the PAX gene were found in subsequent studies. The missense mutation of c.377A>G (p. D126G) and c.55C>T (p.R19W) was reported by Zubkova et al^[9]^ C.92G>T (p.r.31l) missense mutation was reported by Chapla et al^[10]^ in India. So far, the inheritance pattern of MODY9 mutation has been mainly autosomal dominant.

In this study, we report a disease with infantile-onset characterized by autosomal dominant mutation. C.487C>T missense mutation was found in the 7th exon region of the PAX4 gene in the proband, leading to the p.R163W variant in the amino acid sequence, which has not been reported in previous publications. The R163W variation is located in the homologous domain of the PAX4 gene, responsible for the binding of PAX4 protein to the target DNA sequence and associated with conserved amino acid residues between species. R163W variation causes the replacement of polar amino acids by non-polar amino acids, which is suspected to impair the inhibitory effect of PAX4 protein on insulin and glucagon promoters, leading to hyperglycemia and diabetes. Genetic analysis of the family members showed that the father of the patient carried the same heterozygous mutation, while the mother presented with a normal genotype, suggesting that the mutation was autosomal dominant, which is consistent with previous literature reports. The estimated frequency of C.487C>T missense mutation is a low-frequency mutation. According to clinical manifestations and sequencing results, the patient was diagnosed with MODY9. The father of the proband had no diabetes at that time, which may be related to epigenetic factors or incomplete genetic penetrance.

Previous literature showed that the onset age of MODY9 patients ranged from 14 to 50 years. Patients with the same mutation have markedly different clinical presentations, which can manifest as elevated fasting blood glucose levels or impaired glucose tolerance, typical diabetic symptoms in the form of polyuria, polydipsia, wasting, or even ketoacidosis. In the present study, the patient had an early childhood onset and was characterized by typical diabetic symptoms with ketoacidosis. The islet autoantibodies were negative and fasting insulin and C-peptide levels were significantly reduced, which is consistent with the clinical features of MODY9 described previously in the literature.

MODY9 is treated in the same manner as other types of MODY, primarily with a diabetic diet, physical activity, and insulin therapy. Some patients respond to oral hypoglycemic agents and treatment must be adjusted to the progression of the disease. The patient had been diagnosed with diabetes for 17 months at the latest follow-up visit. He was treated with basic and mealtime insulin injections and his blood glucose levels have remained in the normal range since then. The outcome of patients with MODY9 varies significantly. In mild patients, they only present with impaired glucose tolerance, while in severe ones, they may develop renal or retinal complications and even die of end-stage renal failure.

## Acknowledgments

We are grateful to the patient and his family for their participation in this study. We thank Beijing McGinow Technology Co. for their technical support in gene sequencing and data analysis. We would also like to acknowledge the clinical staff of the Endocrinology Department of Beijing Children’s Hospital and the Neonatal Department of Capital Institute of Pediatrics for their assistance in the present study.

## Author contributions

DZ analyzed the clinical data and wrote the manuscript. CC changed the manuscript to the appropriate format. WY, CC, and YS contributed to the review and revision of the manuscript. LR, YP, and YS were involved in the treatment and follow-up of the patient.

**Data curation:** Wenli Yang, Yurong Piao, Li Ren.

**Visualization:** Congli Chen.

**Writing – original draft:** Di Zhang.

**Writing – review & editing:** Congli Chen, Yanmei Sang.
